# Supplementary figures and images for: Adaptation dynamics between copy-number and point mutations
Source: eLife. 2022 Dec 22;11:e82240. doi: 10.7554/eLife.82240 (PMC9833825; doi:10.7554/eLife.82240)

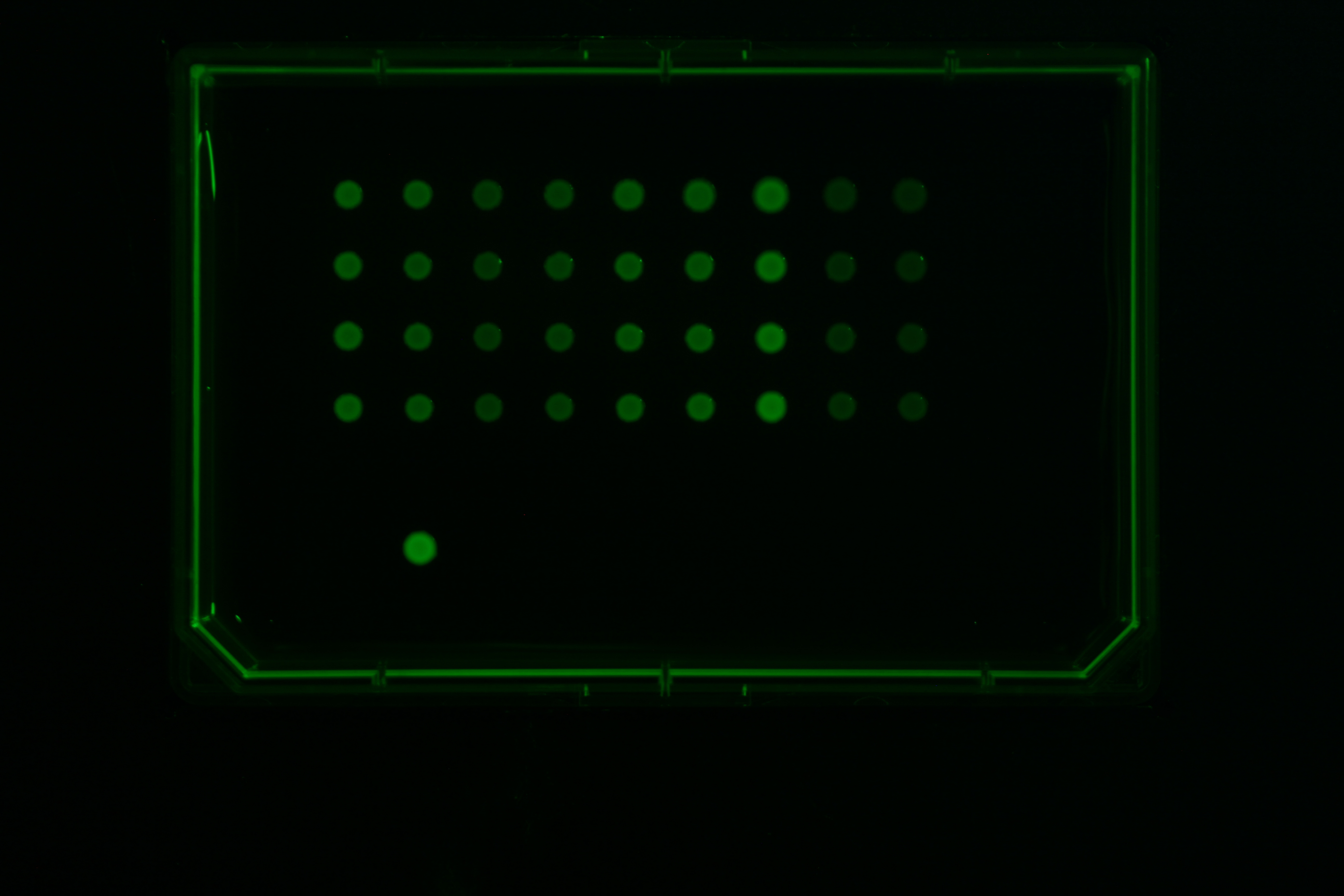

Supplement: Figure 1—figure supplement 1—source data 1. [file elife-82240-fig1-figsupp1-data1.zip › Figure 1 - Figure Supplement 1 - Source Data/for_qPCR-636247363150899708-YFP.jpg]

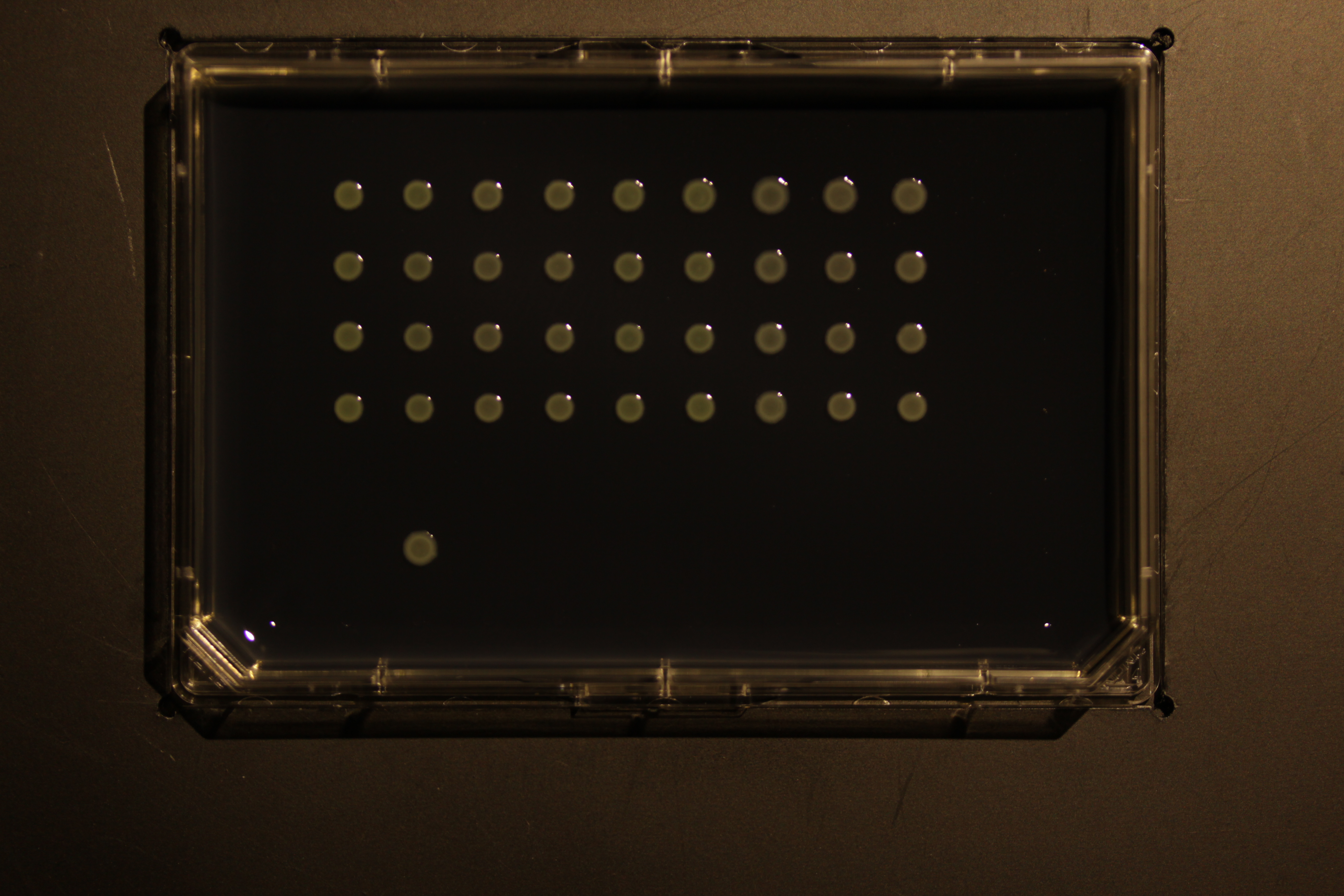

Supplement: Figure 1—figure supplement 1—source data 1. [file elife-82240-fig1-figsupp1-data1.zip › Figure 1 - Figure Supplement 1 - Source Data/for_qPCR-636247363150899708-BF.jpg]

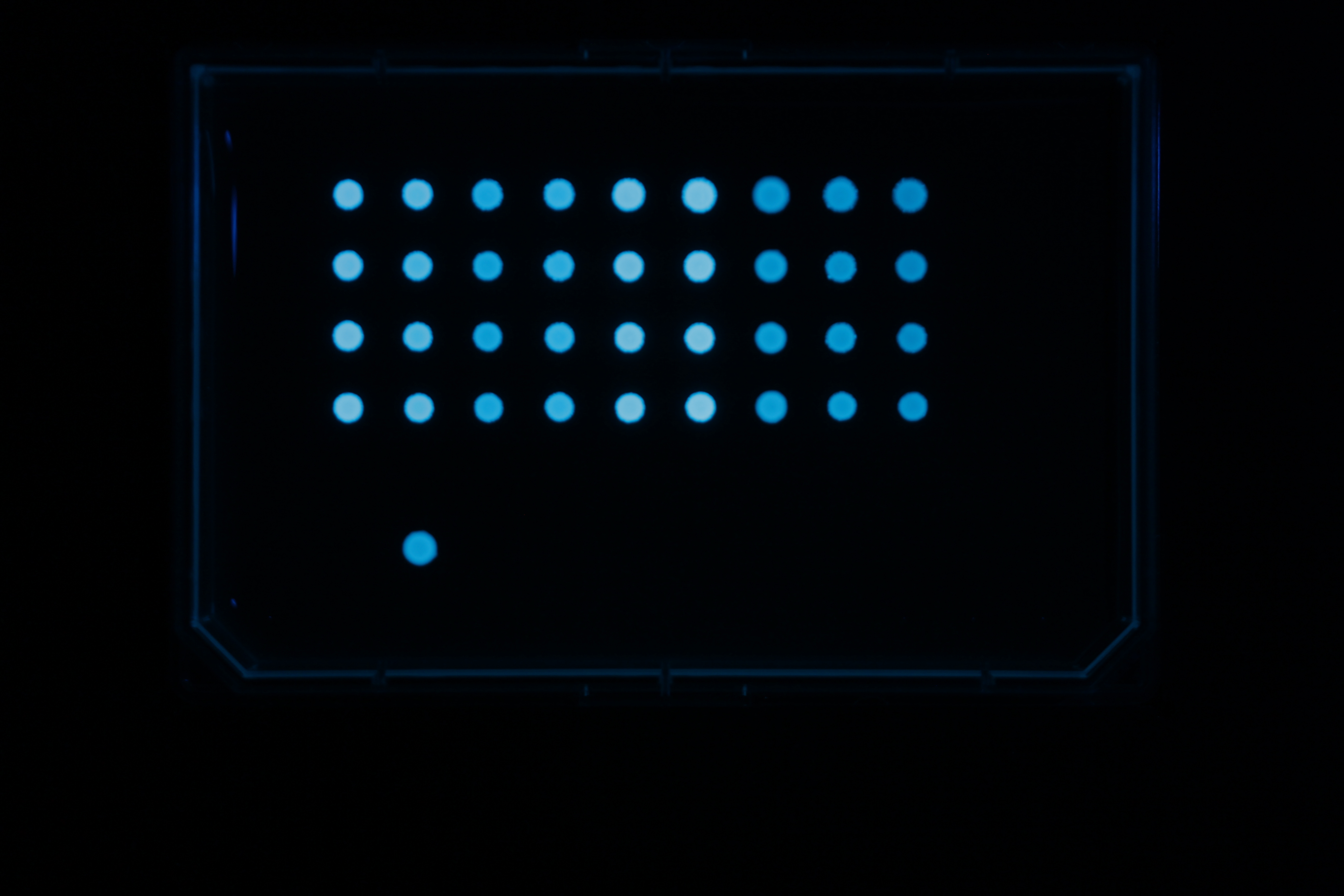

Supplement: Figure 1—figure supplement 1—source data 1. [file elife-82240-fig1-figsupp1-data1.zip › Figure 1 - Figure Supplement 1 - Source Data/for_qPCR-636247363150899708-CFP.jpg]
